# Geoprivacy in Neighbourhoods and Health Research: A Mini-Review of the Challenges and Best Practices in Epidemiological Studies

**DOI:** 10.3389/phrs.2022.1605105

**Published:** 2022-10-27

**Authors:** Ana Isabel Ribeiro, Vasco Dias, Sofia Ribeiro, José Pedro Silva, Henrique Barros

**Affiliations:** ^1^ EPIUnit—Instituto de Saúde Pública, Universidade do Porto, Porto, Portugal; ^2^ Laboratório para a Investigação Integrativa e Translacional em Saúde Populacional (ITR), Porto, Portugal; ^3^ Departamento de Ciências da Saúde Pública e Forenses e Educação Médica, Faculdade de Medicina, Universidade do Porto, Porto, Portugal; ^4^ INESC TEC—Instituto de Engenharia de Sistemas e Computadores, Tecnologia e Ciência, Faculdade de Engenharia da Universidade do Porto, Porto, Portugal; ^5^ Faculdade de Engenharia da Universidade do Porto, Porto, Portugal; ^6^ Instituto de Sociologia da Universidade do Porto, Porto, Portugal

**Keywords:** public health, epidemiology, privacy, confidentiality, neighbourhoods, data protection, anonymization

## Abstract

Neighbourhood and health research often relies on personal location data (e.g., home address, daily itineraries), despite the risks of geoprivacy breaches. Thus, geoprivacy is an important emerging topic, contemplated in international regulations such as the General Data Protection Regulation. In this mini-review, we briefly assess the potential risks associated with the usage of personal location data and provide geoprivacy-preserving recommendations to be considered in epidemiological research. Risks include inference of personal information that the individual does not wish to disclose, reverse-identification and security breaches. Various measures should be implemented at different stages of a project (pre-data collection, data processing, data analysis/publication and data sharing) such as informed consent, pseudo-anonymization and geographical methods.

## Introduction

Tracking the residential, school and workplace locations of individuals can be of utmost importance for health research, as it allows the linkage to environmental exposures and addressing emerging public health concerns and etiology questions (1). Remarkable health benefits have accrued to society from epidemiological research using participant’s location data, such as the identification of environmental hazards, carcinogens, and other modifiable risk factors and infectious disease control.

Epidemiological studies with diverse designs (cohort, case-control, cross-sectional surveys) often collect participants’ location data, which are then linked to local environmental data using Geographic Information Systems (GIS) (2, 3). In addition, epidemiological studies are harnessing the potential of spatial data about individuals collected from embedded sensors, wearables and smartphones to conduct geographic momentary assessments (4). While in the past the collection of geographical data in epidemiological research was mostly completed using questionnaires, today, with GIS and, particularly, with the growing amount of sensor-based data, there is no ceiling for the amount of geographical data that can be collected. Consequently, the privacy and confidentiality of geographic data (geoprivacy) in health research became an important emerging topic (5). Geoprivacy refers to the “individual rights to prevent disclosure of the location of one’s home, workplace, daily activities, or trips” (6). It merges two interrelated concepts: confidentiality and privacy. Confidentiality protects against unauthorised use of information in the possession of an institution, while privacy controls an individual’s right to limit the information that the institution collects, maintains, and shares. From a legal perspective, it relates to two fundamental rights: the right to privacy or private life, enshrined in the Universal Declaration of Human Rights (Article 12), the European Convention of Human Rights (Article 8), the European Charter of Fundamental Rights (Article 7), and the right to data protection, also provided in the European Charter (Article 8).

As personal data quality and quantity increases, so does awareness of personal data protection. This is reflected by the legal architecture of the Treaty on the Functioning of the European Union (TFEU), which provides an obligation to lay down data protection rules for the processing of personal data, and by the General Data Protection Regulation (GDPR), fully applicable in the European Economic Area since 25th May 2018 (7). The GDPR imposes strict rules for the collection, storage, processing and transmission of personal data, charging hefty penalties for privacy violations. It defines personal data as any information about an identified subject or who can be identified through references, such as “a name, an identification number, location data, an online identifier or to one or more factors specific to the physical, physiological, genetic, mental, economic, cultural or social identity of that natural person” (7).

However, current guidelines for epidemiological studies such as the “International Ethical Guidelines for Epidemiological Studies” (8) and similar documents (9) completely overlook geoprivacy-preserving practices. In addition, literature reviews suggest that researchers are not employing appropriate geoprivacy-preserving measures (10). A literature review of 57 studies concluded that only 28% of the articles used geoprivacy-preserving techniques to depict location data (11).

Given this, this mini-review briefly addresses the main risks associated with the usage of personal location data in epidemiological studies, and provides concrete geoprivacy-preserving recommendations.

## Methods

To identify the potential risks associated with the usage of personal location data, we searched PubMed on 14 April 2022, for articles published from years 2000–2022 with titles that included the search terms: (“geographic” or “spatial” or “location”) and (“privacy” or “confidentiality” or “geoprivacy”): 63 references were identified. In addition, we searched for evidence within grey literature sources using Google.com and Google Scholar. The exclusion criteria were: reports/articles for which full text was not available or were not written in English, Portuguese, Spanish, German, French, or Italian. From the reports and articles selected, additional references were identified by a manual search among the cited references. We limited our search to strictly titles, as this study is structured as a non-systematic mini-review and does not intend to follow a systematic method of study selection nor to be reproducible and exhaustive (12). In total, 21 articles were deemed pertinent for this article.

To provide concrete geoprivacy recommendations to be used in epidemiological investigation, we considered the GDPR and the guidelines identified in the included studies. We focused on four chronologically ordered categories of recommendations: pre-data collection, data processing, data analysis and publication, and data sharing with third parties.

## Results

### Risks Associated With the Usage of Personal Location Data

The disclosure of locations may violate geoprivacy when it is used or allowed to infer personal attributes (e.g., deprivation levels of neighbourhood, crime rates) or membership information (e.g., neighbourhood, workplace) based on the linkage with other public or non-public datasets (13). Equally important, geoprivacy can be breached if the disclosed locations reveal information that the individual never intended or agreed to share (14, 15), including sensitive spatial data, when allowing for the inference of certain aspects of individuals’ private life (e.g., religion, sexual orientation), which can be inferred when tracking individual’s itineraries. Such issues are particularly relevant for epidemiological studies, as participants’ locations are coupled with a vast amount of identifiable and sensitive clinical, genetic, social, and economic data (10).

For instance, the disclosure of the residential location of HIV or tuberculosis cases in a small neighbourhood may lead to neighbourhood stigmatisation and disruption of social dynamics due to feelings of fear and distrust. Similarly, the disclosure of the residential location of wealthy individuals may put them at unexpected risk of robbery.

Re-identification through linking of several datasets is also possible (including publicly available data) (16). Suppose one has access to two separate datasets, which separately do not contain details that allow identification of individuals—one about the residence of participants by census tract and a publicly available dataset with the locations of domestic violence crimes at the same geographical level. With these two datasets, one may potentially identify if a participant was involved in a crime.

The risk of geoprivacy breaches also increases when the dataset containing information on individuals, such as addresses and daily activities and trips, is collected or processed in smartphones connected to multiple sensors (e.g., cameras, microphones) and by online service providers such as commercial smartphone applications (apps) or online geocoding services (13, 17). Reviews of popular health apps concluded that frequently a written privacy policy is lacking, policies regarding third party transmission are omitted, or the legal jurisdictions that would handle data are not specified (18, 19). In addition, many institutions cannot perform geocoding/geoprocessing activities and rely on external organizations. Even if allowed by legal or regulatory framework, sharing locational data with third-parties may increase the risk of geoprivacy breaches.

Also, whenever security and data governance frameworks are not checked and put in place previously, a research project is more likely to be at risk of personal data breaches through data theft, data loss, data disclosure to non-authorised parties and other unwanted intrusions (10). Generally, the number and severity of data breaches has increased as a result of digitalization and increased connectivity (20), and is currently a matter of concern (21). Data breaches often involve avoidable human errors (21), which means they are more than a technical issue. In fact, they might be related to technological factors, but also to organisational and management-related, human, and regulatory/auditing factors (20). Therefore, preventive measures might be implemented at different levels (20). In scientific research, data security is frequently absent of the discussions about confidentiality and privacy (13, 22), therefore, the risks of data theft, data loss and unauthorised access are likely neglected (13).

At the publication stage, authors and publishers should also work to protect personal location data (23). Often, research papers present point maps of participants’ locations to illustrate geographical patterns of health events, but mapped locations can be reverse-engineered into actual locations, disclosing private information (10, 11, 24–26). A review of 19 papers published between 1994 and 2005 showed that a reverse-identification method was able to identify 79% of the patients’ home locations (25).

### Recommendations

Within this context, geoprivacy protection measures, in compliance with the applicable regulations, must be put into practice during the different phases of a project. We will describe concrete recommendations to be implemented within the scope of epidemiological studies, considering, in particular, the EU legal framework. These are summarized in Table 1.

**TABLE 1:** Summary of the geoprivacy-preserving recommendations in neighbourhoods and health research.

Pre-data collection	Data processing	Data analysis and publication	Data sharing with third parties
A data governance framework, guided by the data minimization principle, must be established	In-house georeferencing and geoprocessing should be preferred	Researchers must only access derived environmental exposures	Geographical coordinates, exact addresses or complete postcodes should not be provided under any circumstances, only derived environmental exposures, pseudo geographical codes and geomasked data
Informed consent from the participants must be collected	GIS analyst should only receive pseudoanonymised data (identifiers and quasi-identifiers must be removed) using encrypted files	If geographical information is needed, the geographical unit code should be substituted by pseudo-codes	If external institutional are contracted for georeferencing/geoprocessing, a Data Processing Agreement must be celebrated
Transparent and detailed location data processing protocol should be created	Usage of secure computers and techniques during georeferencing and exposure assessment	If exact location data is needed (e.g., coordinates, addresses) the researcher should provide detailed information on why the requested geography is needed and how geoprivacy will be safeguarded (a DPIA might be required)	
Data Protection Impact Assessment (DPIA) may be required	Coordinates and exposure variables should be sent back to the database administrator using encrypted files and secure channels	Mapping the actual locations of participants should be avoided in publications and other dissemination means	
Data Management Plan (DMP) guided by the FAIR principles must be created	Specifically purchase and use wearable devices for the study	If mapping is needed, geomasking techniques must be implemented	
Members of the project should be either trained or experts in geoprivacy threats			

To generate this list of recommendations, we considered the General Data Protection Regulation (GDPR) and the guidelines identified in the included studies (*n* = 21).

#### Pre-Data Collection

During design, whenever consent is used as the legal basis for processing, the informed consent of the participant agreeing with the processing of location data shall be obtained before data collection begins while complying with GDPR specific information requirements (Article 13). A clear data governance framework must be established before data collection, where, following a privacy by design approach, issues such as criteria for data sharing with third parties, levels of access, storage periods and informed consent clarity should be discussed, and it should be ensured that the institution establishes secure measures to prevent geoprivacy breaches (13). This implies that the members of the project should be either trained or experts in geoprivacy threats. The data minimisation process should guide the entire data governance model, i.e., the amount of collection of personal information should be limited to what is directly relevant and necessary to accomplish a specified purpose (Article 5 of the GDPR).

Particularly, it is paramount to develop a transparent and detailed location data processing protocol in conformity with data protection principles. Data processing must be lawful and transparent to participants, and a communication strategy shall be designed considering the intended groups of participants. Overall, the general principles of effectiveness, necessity, and proportionality must guide any measure. To minimize security breaches, Kounadi and Resch recommend the appointment of a “privacy manager”, who should implement a range of data security measures. These include training data processors and controllers, ensuring technical security measures, and ensuring that the research design is as privacy unintrusive as possible (13).

At this stage, a Data Protection Impact Assessment (DPIA)—a process designed to systematically describe personal data processing operations and to identify and minimise risks—may be legally required or, at least, act as a recommended good practice to assess geoprivacy risks, and determine suitable technical and organisational measures to reduce them to acceptable levels. The Data Protection Officer (DPO) should be consulted throughout the course of the DPIA and any other key stakeholders involved in the project. A DPIA is a living document that should be revised and updated whenever required during the project implementation.

A Data Management Plan (DMP) may also be helpful with respect to the mapping of the life cycle of the data, documenting the defined data collecting, access and sharing procedures, as well the data retention periods, while addressing data security and ethical concerns. Data management should follow the FAIR guidelines, which means that data should be findable, accessible, interoperable and re-usable (27).

#### Data Processing

According to the GDPR, processing includes all operations performed upon personal data, including collection, linkage, storage or deletion (Article 2, no. 2). At any event, following data collection and during the whole duration of the project, appropriate safeguards must be in place, e.g., the database administrator must store the location data in a safe computer and database, using encryption and protected networks or by implementing adequate access controls.

When conducting address georeferencing and data linkage to geographical units and local environmental data, the database administrator should provide the researchers who must access the data (usually spatial data analysts working at the institution) the pseudonymised location data using encrypted files. Identifiers or quasi-identifiers must be removed. The GDPR highlights pseudonymisation and anonymisation of data as key elements, in articulation with the data minimisation principle (“data should be anonymised or deleted once it loses utility for the purposes of the research”) (Article 89, no. 1). Data is to be considered as pseudonymised when it can only be attributed to specific individuals in combination with additional identifiable data, which is kept securely separated by the data controller or another data processor, for instance, using a key (e.g., participant ID) as a pseudonym. Procedures such as address georeferencing and linkage to environmental exposures and geographical areas should be conducted using secure computers and techniques (28).

In-house address georeferencing and geoprocessing is preferable and, if an external institution is contracted (in case the data governance framework allows it), full trust is needed in the capabilities of the external institution to conduct accurate georeferencing, and to destroy the address data afterwards (16). For these cases, a Data Processing Agreement must be celebrated.

Afterwards, the coordinates obtained through address georeferencing and the derived exposure variables should be sent back to the database administrator using encrypted files and secure channels. At this stage, the controller will be able to reverse the pseudonym back to the identifiable data.

As mentioned before, special care must be taken in epidemiological studies using apps that operate on smartphones that may have access to other apps and sensors (e.g., microphone, camera), as security risks are harder to assess. It is recommended to purchase and use wearable devices which permit the data controller to determine how and in which systems data is processed and stored, in an encrypted form (13).

#### Data Analysis and Publication

Often researchers do not require the actual residential location of participants, but an indication about the unit of aggregation for multi-level analysis (29). For that, the real geographical unit code (e.g., official code of a census tract, parish, municipality) should be substituted by pseudo-codes that allow to identify that a group of study participants reside within the same neighbourhood, but not where the neighbourhood is actually located or to conduct record linkage with other spatial datasets.

If location data is needed—e.g., for spatially explicit studies or for measuring additional environmental exposures—the researcher should provide detailed information on why the requested geography is needed and how geoprivacy will be safeguarded. If, for any reason, a DPIA was not carried out to this point or did not mention the need for this location data, the DPO may be consulted to assess the possible privacy threats and the institutional review board may deny access to that data if geoprivacy is at risk.

Finally, at the stage of publication and map creation, it is important to avoid mapping the actual point locations of participants, being advisable to geographically mask sensitive data. Geomasking is a process of encoding the geography of records to protect participants’ geoprivacy, while still allowing to accurately characterise the spatial distribution of events and run valid geographical analyses (30). Armstrong, Rushton, and Zimmerman provided a comprehensive summary of various geomasking techniques, including affine transformation, random perturbation, spatial aggregation, and point aggregation (31).

#### Data Sharing With Third Parties

The GDPR enables the free-flow of personal data across EU member states and other countries that are deemed to provide adequate data protection standards. Cohort data is frequently accessed by multiple researchers inside and outside an institution.

When providing location data to third parties, it may be necessary, or advisable, to remove some elements, always considering a data minimisation approach. Geographical coordinates, exact addresses or complete postcodes should not be provided under any circumstances, only derived environmental exposures (e.g., levels of pollution, distance to green space), pseudo geographical codes and geomasked data.

If external institutions are contracted for georeferencing/geoprocessing, a Data Processing Agreement must be celebrated.

## Conclusion

Today, the increasing amount of detailed location data and powerful new tools opened large avenues to study the influence of the local neighbourhood environment on health. Yet, institutions and researchers must be aware of the breaches in geoprivacy that may occur, and thus, work to implement strategies compliant with the GDPR or other country-specific data protection regulations. For an effective implementation of geoprivacy-preserving strategies, a constant interdisciplinary dialog between the data protection officers, ethicists, researchers, and study participants is desirable.

## Abbreviations

EU, European Union; GDPR, General Data Protection Regulation; GIS, Geographic Information Systems; DMP, Data Management Plan; DPIA, Data Protection Impact Assessment; DPO, Data Protection Officer; TFEU, Treaty on the Functioning of the European Union.
